# Calpain-1 deletion impairs mGluR-dependent LTD and fear memory extinction

**DOI:** 10.1038/srep42788

**Published:** 2017-02-16

**Authors:** Guoqi Zhu, Victor Briz, Jeff Seinfeld, Yan Liu, Xiaoning Bi, Michel Baudry

**Affiliations:** 1 Graduate College of Biomedical Sciences, Pomona, CA 91766, USA; 2 Key Laboratory of Xin’an Medicine, Ministry of Education, Anhui University of Chinese Medicine, Hefei 230038, China; 3 VIB Center for the Biology of Disease, KU Leuven, 3000 Leuven, Belgium; 4 College of Osteopathic Medicine of the Pacific Western University of Health Sciences Pomona, CA 91766, CA 91766, USA.

## Abstract

Recent studies indicate that calpain-1 is required for the induction of long-term potentiation (LTP) elicited by theta-burst stimulation in field CA1 of hippocampus. Here we determined the contribution of calpain-1 in another type of synaptic plasticity, the long-term depression (LTD) elicited by activation of type-I metabotropic glutamate receptors (mGluR-LTD). mGluR-LTD was associated with calpain-1 activation following T-type calcium channel opening, and resulted in the truncation of a regulatory subunit of PP2A, B56α. This signaling pathway was required for both the early and late phase of Arc translation during mGluR-LTD, through a mechanism involving mTOR and ribosomal protein S6 activation. In contrast, in hippocampal slices from calpain-1 knock-out (KO) mice, application of the mGluR agonist, DHPG, did not result in B56α truncation, increased Arc synthesis and reduced levels of membrane GluA1-containing AMPA receptors. Consistently, mGluR-LTD was impaired in calpain-1 KO mice, and the impairment could be rescued by phosphatase inhibitors, which also restored Arc translation in response to DHPG. Furthermore, calpain-1 KO mice exhibited impairment in fear memory extinction to tone presentation. These results indicate that calpain-1 plays a critical role in mGluR-LTD and is involved in many forms of synaptic plasticity and learning and memory.

Learning and memory are widely considered to result from changes in connectivity within neuronal circuits. In particular, two forms of activity-dependent synaptic plasticity in hippocampus, long-term potentiation (LTP) and long-term depression (LTD) of synaptic transmission have been proposed to represent cellular models of various forms of learning and memory^1^,^2^,^3^. While LTP has been shown to be involved in fear conditioning, LTD and depotentiation have been proposed to participate in the extinction of the fear response^4^,^5^,^6^,^7^, which takes place when the context/tone stimulus is repeatedly presented in the absence of electric shock. However, there is still an animated debate concerning whether extinction represents erasure of an existing memory, or new learning^8^,^9^. Because of the relevance of this mechanism for the treatment of patients with traumatic memories, there has been considerable interest in understanding the cellular/molecular mechanisms underlying the extinction phenomenon.

Various cellular cascades have been shown to be triggered by different stimulation protocols used to induce LTP, all of which converge to produce identical changes in structure and function of dendritic spines at glutamatergic synapses, namely increased number of AMPA receptors and increased size of dendritic spines^10^. Similarly, various forms of LTD result from the activation of different types of glutamate receptors leading to a decreased number of AMPA receptors and possibly smaller dendritic spines^11^,^12^,^13^. Some of the signaling pathways required for LTP induction or expression, such as those mediated by PI3K/Akt or ERK, are known to be involved in LTD as well^14^,^15^. The activation of similar intracellular signaling cascades leading to either potentiation or depression indicates that additional upstream or downstream cascades of these pathways are important for triggering either potentiation or depression.

It has long been known that changes in intracellular calcium concentration are required for both LTP and LTD^16^,^17^. Previous studies have implicated voltage-dependent calcium channels (VDCC) and inositol triphosphate (IP_3_) receptors in metabotropic glutamate receptor-dependent LTD (mGluR-LTD)^18^,^19^. While one study showed that postsynaptic application of calcium chelator failed to block mGluR-LTD^20^, the experiments were done in hippocampal slices from neonatal animals. A later study showed the existence of a developmental switch in the synaptic mechanisms of mGluR-LTD, indicating that in slices from more mature animals, mGluR-LTD is associated with protein synthesis-dependent decrease in postsynaptic AMPA receptors^21^. Although no studies have directly shown that postsynaptic calcium plays a role in mGluR-LTD in slices from adult rats or mice, CamKII activation has been shown to be involved^22^. It is currently unclear how mGluR activation is linked to downstream signaling cascades during mGluR-LTD. Recent findings indicate that the calcium-activated proteases, calpains, are critical regulators of LTP and memory formation^23^. In particular, while calpain-1 activation is required for LTP formation^24^, calpain-2 activation limits the extent of potentiation during the consolidation period^25^. Nevertheless, whether calpain and associated signaling pathways participate in LTD has not yet been addressed.

In this study, we examined the role of calpain-1 in LTD in hippocampal Schaffer collateral-CA1 synapses. While NMDA receptor-dependent LTD (NMDAR-LTD) was normal in calpain-1 knock-out (KO) mice, mGluR-LTD and fear extinction were impaired. Furthermore, we identified a novel role for the regulatory subunit of protein phosphatase 2A (PP2A) B56α in synaptic plasticity, as its degradation by calpain-1 was found to be required for mGluR-mediated translational control and LTD.

## Results

### Calpain-1 deletion impairs mGluR- but not NMDAR-dependent LTD

**Figure 1:**
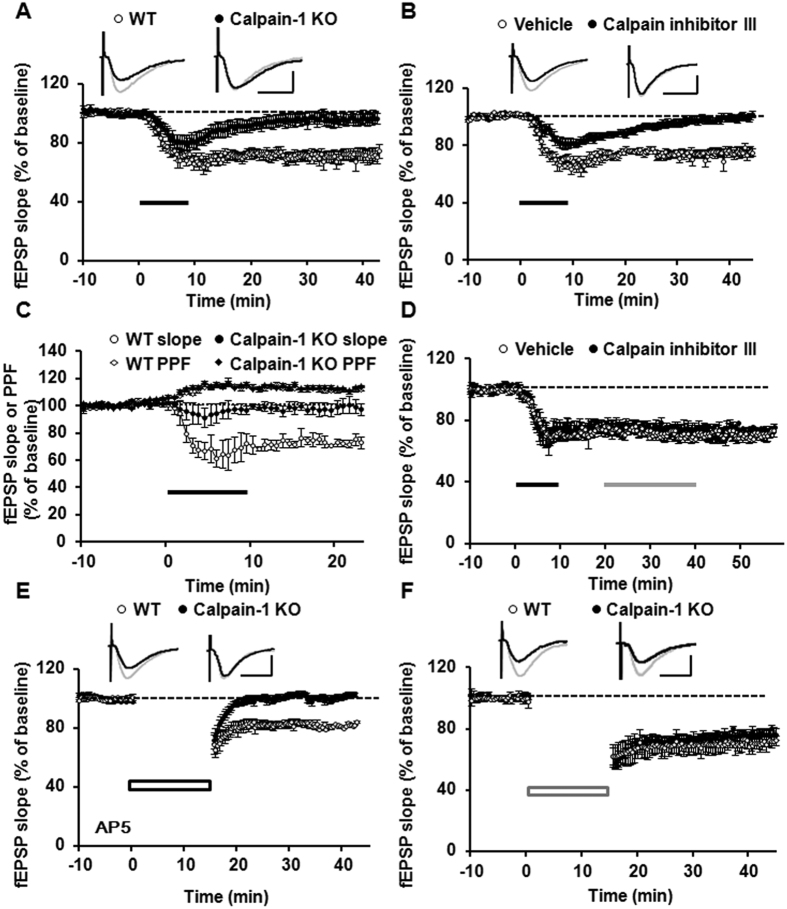
Calpain-1 deletion impairs DHPG-induced LTD. (**A**) DHPG application (100 μM for 10 min, horizontal bar) resulted in LTD in field CA1 of WT mice but the slopes of field excitatory postsynaptic potential (fEPSP) returned to baseline within 10 min in slices from calpain-1 KO mice. (**B**) Application of calpain inhibitor III (10 μM) during DHPG application inhibited DHPG-induced LTD in hippocampal slices from WT mice. (**C**) DHPG-induced enhancement of paired pulse facilitation (PPF) was not affected in hippocampal slices from calpain-1 KO mice. (**D**) Application of calpain inhibitor III (10 μM) 10 min after DHPG treatment did not modify mGluR-LTD. (**E**) LTD elicited by PP-LFS in the presence of AP5 (50 μM) was also impaired in calpain-1 KO mice. (**F**) On the other hand, LFS-induced LTD was not affected in hippocampal slices from calpain-1 KO mice, and was identical to that in slices from WT mice. Results are means ± S.E.M. of 5–6 slices from 4 different animals. Grey and black traces represent responses during baseline and 40 min after DHPG application, respectively. Scale bar: 0.5 mV/10 ms.

We previously showed that calpain-1 activation following NMDA receptor stimulation was necessary for theta burst stimulation (TBS)-induced LTP^24^. However, the role of calpains, and in particular calpain-1, in LTD has not yet been investigated. In this study, we used calpain-1 KO mice to determine the role of calpain-1 in various forms of LTD at the Schaffer collateral synapses in field CA1 of acute hippocampal slices. LTD was induced by: (i) low frequency stimulation (1 Hz, 15 min, LFS-LTD), (ii) a short application (10 min) of the mGluR1/5 receptor agonist, DHPG (mGluR-LTD) coupled with very low frequency stimulation (0.05 Hz, 10 min), and (iii) low frequency paired-pulse stimulation (50 ms interval, 1 Hz, 15 min) in the presence of the NMDA receptor antagonist, AP5. While DHPG application induced stable LTD in hippocampal slices from WT mice, it induced only a short-term depression in slices from calpain-1 KO mice, and responses rapidly returned to baseline values (Fig. 1A). To confirm the role of calpain in mGluR-LTD, we applied a broad spectrum calpain inhibitor, calpain inhibitor III, which inhibits both calpain-1 and calpain-2, before DHPG application in slices from WT mice. Under this condition, DHPG only induced a short-term depression (Fig. 1B). To further characterize the role of calpain-1 in mGluR-LTD, we determined the effect of calpain-1 deletion on paired-pulse facilitation (PPF) before and after mGluR-induced LTD. PPF was similarly increased after DHPG application in WT and calpain-1 KO mice (Fig. 1C). Application of calpain inhibitor III after mGluR-induced LTD had no effect on established LTD (Fig. 1D), suggesting that calpain activation takes place early and for a short period of time during mGluR-LTD.

A second form of mGluR1/5-dependent LTD was induced by using paired-pulse stimulation delivered at low frequency (PP-LFS), in the presence of an NMDA receptor antagonist, AP5^26^. Under these conditions, PP-LFS induced LTD in hippocampal slices from WT mice but not calpain-1 KO mice (Fig. 1E). Finally, NMDAR-LTD was induced using LFS (1 Hz for 15 min). This type of LTD was not affected by calpain-1 deletion (Fig. 1F).

### DHPG-induced stimulation of Akt/mTOR/S6 pathway is deficient in calpain-1 KO mice

**Figure 2:**
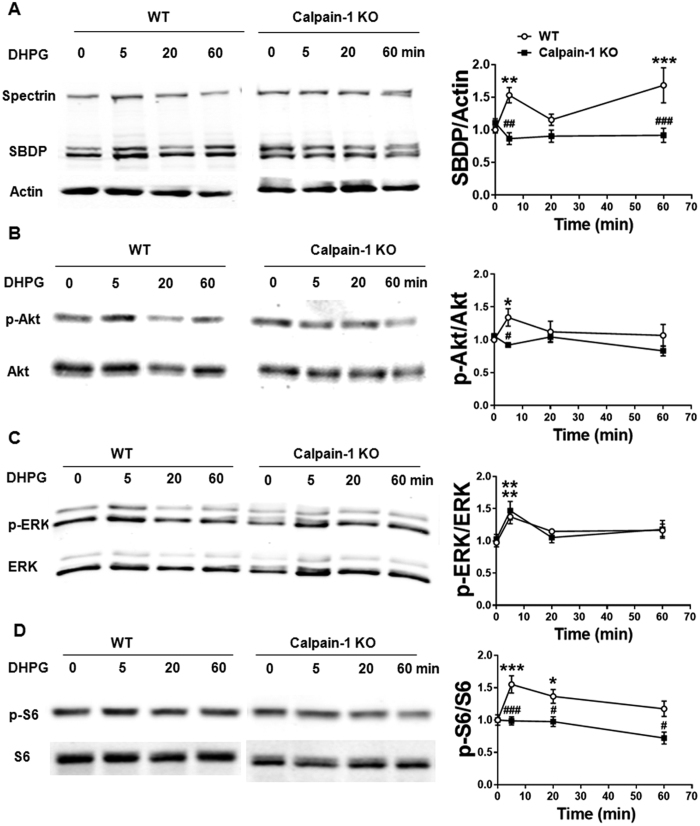
DHPG application triggers calpain-1 activation and Akt and S6 phosphorylation. Hippocampal slices were treated with DHPG (100 μM, for 10 min). Slices were collected at various times after DHPG application, homogenized, and aliquots of the homogenates were processed for western blots labeled with the indicated antibodies, as described in Materials and Methods. (**A–D**) Left panel: Representative blots of spectrin and spectrin breakdown products (SBDP) (**A**), phospho-Akt (pAkt) and Akt (**B**), p-ERK and ERK (**C**), p-S6 and S6 (**D**) after DHPG application in hippocampal slices from WT and calpain-1 KO mice. Right panel: Quantification of the ratios of the indicated proteins after DHPG application in WT and calpain-1 KO mice. In all cases, results are means ± S.E.M. of 4–7 slices from 4 different animals; *p < 0.05, **p < 0.01, ***p < 0.001, as compared with baseline values (time 0), ^#^p < 0.05, ^##^p < 0.01, ^###^p < 0.001, as compared with the corresponding time point in WT mice (Two-way ANOVA + Bonferroni test).

The above results suggested that calpain-1 was activated during mGluR-LTD. To directly address this question, we evaluated calpain activity after DHPG application in hippocampal slices from WT and calpain-1 KO mice by analyzing the formation of calpain-specific spectrin breakdown products (SBDP) by western blots. In slices from WT mice, DHPG induced an increase in SBDP as early as 5 min after DHPG application. SBDP levels returned to baseline by 20 min, but a later increase was evident 60 min after DHPG (Fig. 2A). In contrast, SBDP levels were not affected by DHPG application at any of the time points tested in hippocampal slices from calpain-1 KO mice (Fig. 2A). These results confirm that mGluR-LTD is associated with calpain-1 activation in hippocampal slices.

We next determined the effects of DHPG on Akt and ERK signaling pathways, both of which have been shown to be required for mGluR-LTD^14^,^15^,^27^. DHPG induced a rapid and transient increase in Akt phosphorylation (p-Akt) in hippocampal slices from WT mice but not calpain-1 KO mice (Fig. 2B). In contrast, DHPG caused a short-term activation of ERK both in WT and in calpain-1 KO mice (Fig. 2C). These results indicated that DHPG stimulated Akt but not ERK phosphorylation required calpain-1. We also determined the effects of DHPG on mTOR phosphorylation, which is downstream of Akt activation^15^. mTOR phosphorylation remained unchanged following DHPG treatment at 5, 20 and 60 min after DHPG application in both WT and calpain-1 KO mice (data not shown). This result seems to be in conflict with previous findings^15^, but the modest increase in mTOR phosphorylation previously reported along with its very rapid dephosphorylation after DHPG washout^28^,^29^ could have been easily missed under our experimental conditions. Alternatively, the different DHPG concentrations used or the fact that we used whole hippocampal slices instead of the CA1 region might also account for the differences between the studies.

Several translation factors have been implicated in mGluR-LTD, including eukaryotic initiation factor alpha (eIF2α) and ribosomal S6 protein^28^,^30^. We determined the effects of DHPG on eIF2α and S6 phosphorylation at different time points in hippocampal slices from WT and calpain-1 KO mice. A transient increase in phospho-S6 levels was found 5 and 20 min after DHPG application in WT mice, and phospho-S6 levels returned to baseline by 60 min (Fig. 2D), in good agreement with previous findings^28^. In contrast, DHPG failed to increase S6 phosphorylation in calpain-1 KO mice at any of the time points tested (Fig. 2D). eIF2α phosphorylation remained unchanged following DHPG treatment in hippocampal slices from both WT and calpain-1 KO mice (Fig. S1).

### DHPG-induced stimulation of Arc translation and LTD involve calpain-1-mediated PP2A inactivation

We next seeked to identify the calpain substrate(s) linking calpain-1 activity to stimulation of the Akt signaling pathway. PHLPP1α and β (also referred to as SCOP) are phosphatases, which negatively regulate Akt and ERK activities, respectively, and they are both calpain-1 substrates^25^,^31^. However, we did not find changes in any of these two proteins after DHPG application, regardless of the genotype (Fig. S1). Phosphatase and tensin homolog (PTEN) is another important negative regulator of Akt, and it is also a calpain-2 specific substrate^32^. Consistent with this notion, PTEN levels were not affected by DHPG treatment (Fig. S1).

**Figure 3:**
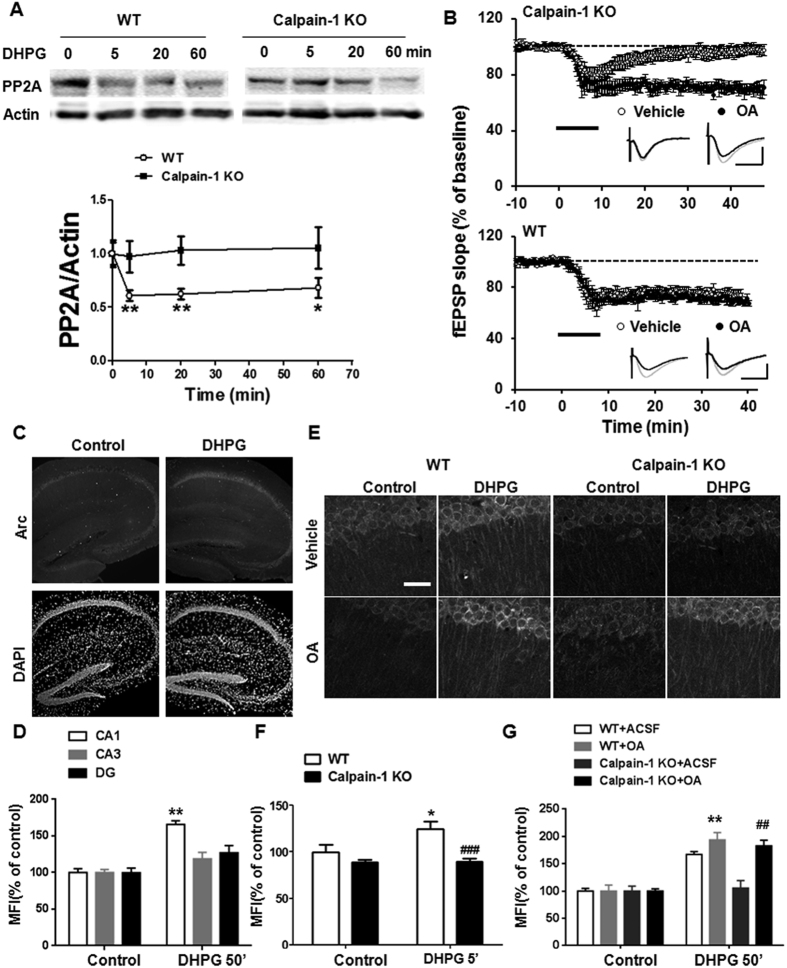
DHPG application results in calpain-1-mediated B56α degradation and PP2A inhibition restores DHPG-induced Arc translation and LTD in calpain-1 KO mice. (**A**) DHPG application in hippocampal slices from WT mice but not from calpain-1 KO mice elicited a rapid decrease in B56α levels. Upper panel: representative blots for B56α after DHPG application in both of WT and calpain-1 KO mice. Bottom panel: quantification of B56α/actin ratio. Results are means ± S.E.M. of 6–9 slices from 4 different animals; *p < 0.05, **p < 0.01, as compared with baseline value (time 0) in WT mice; ^#^p < 0.05, as compared with corresponding time point in WT mice (Two-way ANOVA + Bonferroni test). (**B**) The PP2A inhibitor okadaic acid (OA, 50 nM) restored mGluR-LTD in hippocampal slices from calpain-1 KO mice (top), but did not affect mGluR-LTD in slices from WT mice (bottom). Grey and black traces represent responses during baseline and 40 min after DHPG application, respectively. Scale bar: 0.5 mV/10 ms. Results are means ± S.E.M. of 5–8 slices from 5 different animals. (**C**) In WT mice, DHPG application (100 μM for 10 min) induced a significant increase in Arc levels in CA1 region of hippocampus but not in CA3 or dentate gyrus (DG) at 50 min after DHPG application. Upper panel shows representative images of Arc immunostaining. Bottom panel shows the images of DAPI staining. (**D**) Quantification data of the mean fluorescence intensity (MFI) in different regions of hippocampus; **p < 0.01, as compared with baseline level in WT mice (Two-way ANOVA + Bonferroni test). (**E**) DHPG induced a late (50 min after DHPG) increase in Arc immunostaining in hippocampal slices from WT mice, which was restored by okadaic acid (OA) application in calpain-1 KO mice. (**F,G**) Quantification data of the MFI of Arc immunostaining (somatic and dendritic) after treatment with DHPG for 5 (**F**) or 50 min (**G**). (**F**) *p < 0.05 compared with control in WT mice. ^###^p < 0.001 compared with DHPG in WT mice (Two-way ANOVA + Bonferroni test). (**G**) **p < 0.01 compared with DHPG in WT mice. ^##^p < 0.01 compared with DHPG in calpain-1 KO mice (Two-way ANOVA + Bonferroni test). In all cases, results are means ± S.E.M. of 5–10 slices from 5 different animals.

Protein phosphatase 2A (PP2A) dephosphorylates Akt^33^,^34^, and the PP2A regulatory subunits, B56α and B56γ, are calpain-1 substrates^35^. Thus, we tested whether B56α levels were altered during mGluR-LTD. As shown in Fig. 3A, levels of B56α were significantly decreased after DHPG application in hippocampal slices from WT mice at all the time points tested (P = 0.0019; One-way ANOVA). In contrast, B56α levels remained unchanged after DHPG application in hippocampal slices from calpain-1 KO mice. These results confirmed that B56α is also a calpain-1 substrate in neurons, and that it is truncated during mGluR-LTD. The degradation of B56α by calpain-1 after DHPG treatment is expected to decrease PP2A activity, which could contribute to Akt activation and LTD. To confirm this hypothesis, we tested whether a phosphatase inhibitor with high selectivity for PP2A, okadaic acid^36^, could reverse mGluR-LTD impairment in calpain-1 KO mice. As predicted, pre-application of okadaic acid (50 nM) completely restored DHPG-induced LTD in hippocampal slices from calpain-1 KO mice (Fig. 3B). These results were reproduced with a structurally different PP2A inhibitor, calyculin-A (10 nM) (Fig. S2). In contrast, neither PP2A inhibitor affected DHPG-induced LTD in hippocampal slices from WT mice (Fig. 3B).

The activity-regulated cytoskeletal protein, Arc, is rapidly synthesized in response to DHPG application^37^,^38^. While several studies have implicated various proteins capable of regulating early Arc translation during mGluR-LTD, including eukaryotic elongation factor-2 kinase (eEF2K) and Fragile-X mental retardation protein (FMRP)^37^,^39^, those involved in the late phase of Arc synthesis are largely unknown. Thus, we determined the effects of DHPG application on both the early and late phase of Arc translation in hippocampus. In hippocampal slices from WT mice, Arc expression was increased in CA1 region 5 and 50 min after DHPG application (Figs 3E and S3). No significant effects were found in CA3 or dentate gyrus (Fig. 3C,D), a result in agreement with recent findings from our laboratory in rat hippocampus^13^. In contrast, Arc expression was not altered by DHPG application in hippocampal slices from calpain-1 KO mice at any of the time points tested. Notably, treatment with okadaic acid restored late Arc translation in response to DHPG in hippocampal slices from calpain-1 KO mice (Fig. 3E,G). Treatment with okadaic acid further increased Arc levels in WT mice, as compared to DHPG alone, indicating that DHPG-induced Arc translation is not saturated during mGluR-LTD. High magnification images indicated that Arc was present in PSD95-positive puncta, consistent with dendritic spine localization (Fig. S4). Quantification of the number of puncta in field CA1 indicated that DHPG application increased the number of Arc-positive puncta in slices from WT but not calpain-1 KO mice (Fig. S4B). Incubation of slices with okadaic acid did not significantly modify the number of Arc-positive puncta in slices from WT mice but restored the effect of DHPG in slices from calpain-1 KO mice (Fig. S4B). As activation of mTOR, which is downstream of Akt, has been shown to be required for mGluR-LTD^15^, our results suggested that the Akt/mTOR pathway was involved in DHPG-induced late Arc synthesis. To verify this hypothesis, we incubated hippocampal slices from WT mice with rapamycin (20 nM) before DHPG application. Under these conditions, DHPG-induced LTD was inhibited (Fig. S5A), as previously reported^15^. DHPG-induced late Arc synthesis was also completely abolished (Fig. S5B).

### DHPG application induced a protein synthesis-dependent decrease in GluA1 and increase in PSD95 levels

**Figure 4:**
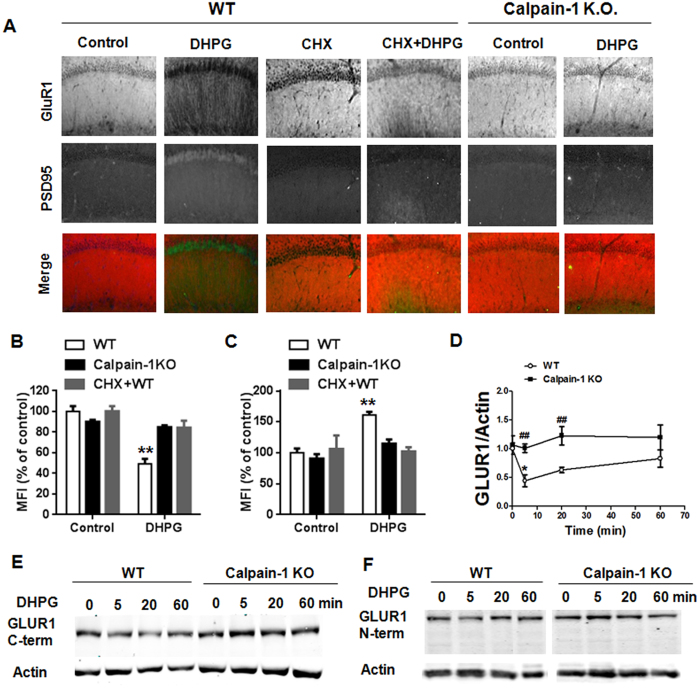
DHPG application induces a protein synthesis-dependent decrease in GluA1 and increase in PSD95. (**A**) Representative images showing that DHPG application (100 μM, for 10 min) causes a remarkable decrease in GluA1 expression and increase of PSD95 in CA1 region of WT mice 50 min after DHPG application, which were blocked by cycloheximide (CHX, 25 μM). In calpain-1 KO mice, DHPG did not affect GluA1 or PSD95 immunostaining. (**B,C**) Quantification data of mean fluorescence intensity (MFI) of GluA1 (**B**) and PSD95 (**C**) immunostaining; **p < 0.01, as compared with the corresponding control (Two-way ANOVA + Bonferroni test). (**D**) Quantification data of GluA1/actin ratio; *p < 0.05, as compared with baseline level; ^##^p < 0.01, as compared with the corresponding time point in WT mice (Two-way ANOVA + Bonferroni test). (**E,F**) Representative blots of GluA1 at various time points after DHPG application in WT and calpain-1 KO mice using a C-terminal (**E**) or an N-terminal (**F**) GluA1 antibody. In all cases, results are means ± S.E.M. of 4–8 slices from 4 different animals.

mGluR-LTD is associated with a sustained decrease in AMPA receptor-mediated synaptic transmission^34^. We thus compared GluA1 expression after DHPG application in hippocampal slices from WT and calpain-1 KO mice by immunohistochemistry. GluA1 expression was markedly decreased 20 min after DHPG application in CA1 region from WT mice (Fig. 4A,B). Conversely, PSD95 immunostaining was increased at the same time point following DHPG application (Fig. 4A,C). The decrease in GluA1 and increase in PSD95 levels induced by DHPG were both inhibited by cycloheximide, a protein translation inhibitor (Fig. 4A–C). Likewise, rapamycin blocked the increase in PSD95 induced by DHPG (Fig. S5B,C). In contrast, DHPG did not affect GluA1 or PSD95 expression in calpain-1 KO mice (Fig. 4A–C). A similar decrease in GluA1 levels was obtained by western blot at different time points after DHPG application in hippocampal slices from WT mice using the same GluA1 C-terminal antibody (Fig. 4D,E).

Calpain has previously been reported to cleave GluA1 in the C-terminal region^40^. Therefore, a different GluA1 antibody (raised against the N-terminal region) was used to rule out the possibility that the decrease in GluA1 observed after DHPG treatment was due to calpain-mediated GluA1 truncation. If this were the case, a truncation would leave intact the N-terminal fragment of GluA1 and a shift in the molecular weight of the GluA1 band would be observed after DHPG application. However, the results were similar to those obtained with the C-terminal antibody and no apparent shift in the GluA1 band was observed (Fig. 4F), which excludes the possibility that DHPG causes GluA1 truncation. These data support the idea that a protein synthesis-dependent decrease in GluA1 levels was involved in the DHPG effect.

### mGluR-LTD is suppressed by a T-type voltage-dependent calcium channel blocker

**Figure 5:**
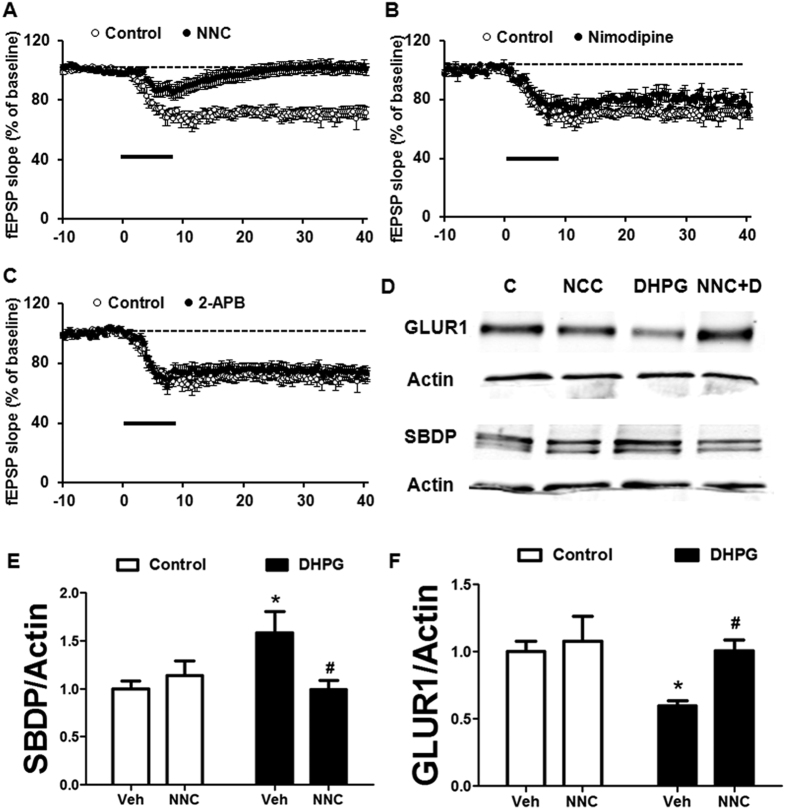
DHPG-induced GluA1 down-regulation and LTD is suppressed by a T-type voltage dependent calcium channel blocker. (**A**) The T-type voltage dependent calcium channel inhibitor NCC (10 μM) blocked DHPG-induced LTD. (**B**) The L-type voltage dependent calcium channel inhibitor nimodipine (10 μM) did not affect LTD. (**C**) The IP3 channel inhibitor 2-APB (50 μM) did not affect DHPG-induced LTD. (**D**) NCC inhibited DHPG-induced increase in SBDP and decrease in GLUA1. (**E,F**) Quantification data of SBDP/actin (**E**) or GluA1/actin (**F**) ratio after 5 min of DHPG treatment in hippocampal slices from WT mice; *p < 0.05, as compared with vehicle control; ^##^p < 0.01, as compared with vehicle DHPG (Two-way ANOVA + Bonferroni test). In all cases, results are means ± S.E.M. of 5–8 slices from 5 different animals.

The above data identified the signaling pathway downstream of DHPG-induced calpain activation. We then addressed the potential calcium source for calpain-1 activation during mGluR-LTD. Both L-type and T-type VDCCs as well as IP_3_ receptors have been previously implicated in mGluR-LTD^12^,^18^. A T-type VDCC blocker, NNC 55-0396 (NCC, 10 μM) (Fig. 5A), but not the L-type VDCC blocker nimodipine (10 μM) (Fig. 5B) or the IP_3_ receptor antagonist 2-APB (50 μM) (Fig. 5C) inhibited DHPG-induced LTD in hippocampal slices from WT mice. Baseline responses were not affected by any inhibitor of these calcium channels (data not shown). Consistent with the electrophysiological data, NCC inhibited DHPG-induced increase in SBDP and decrease in GluA1 (Fig. 5D–F).

### Impaired extinction of fear conditioning in calpain-1 KO mice

**Figure 6:**
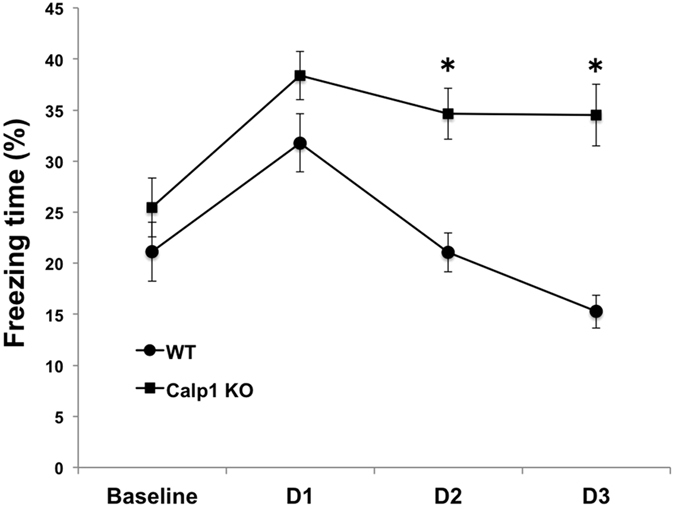
Calpain-1 KO mice exhibit impaired extinction of fear memory. Groups of WT and calpain-1 KO mice were trained in the fear conditioning protocol with 3 tone-shock pairings 1 min apart. Twenty-four h later, they were placed in a different context and exposed to repeated exposure to tone without shock (14 trials with 5 sec tone exposure with an inter-trial interval of 25 sec). This protocol was repeated for 2 more days for a total of 3 days of extinction. The percent of freezing following each tone presentation was determined and the cumulative percent freezing time per day determined and averaged. Results are means ± S.E.M. of 15–20 animals per group. **p < 0.01, as compared with the corresponding time point in WT mice (Two-way ANOVA + Bonferroni test).

We previously reported that calpain-1 KO mice exhibited learning impairment in fear conditioning when trained with one tone-shock pairing but were able to learn when 3 tone-shock pairings were used in the training session^24^,^41^. As LTD has previously been proposed to be involved in extinction^4^,^5^,^6^,^7^, we trained WT and calpain-1 KO mice with 3 tone-shock pairings and exposed them to 3 days of extinction trials with 10 tone presentations for 5 sec every 25 sec. Tone-elicited freezing was determined and values averaged for each of the 3 days of testing. WT and calpain-1 KO mice did not show any difference in the amount of freezing before starting the extinction trials (Fig. 6). However, while WT mice exhibited a typical extinction pattern over the 3 days of testing, calpain-1 KO mice exhibited very little extinction over the same period (Fig. 6).

## Discussion

We recently reported that LTP induced by TBS was impaired in calpain-1 KO mice, whereas high-frequency stimulation-induced LTP was not affected^24^. These findings underscore the fact that calpain-1 is selectively activated by different patterns of electrical activity resulting in different types of synaptic plasticity. In the present study, we identified a novel role for calpain-1 in mGluR- but not NMDAR-LTD, providing additional evidence for the specific involvement of calpain-1 in certain forms of hippocampal synaptic plasticity.

**Figure 7:**
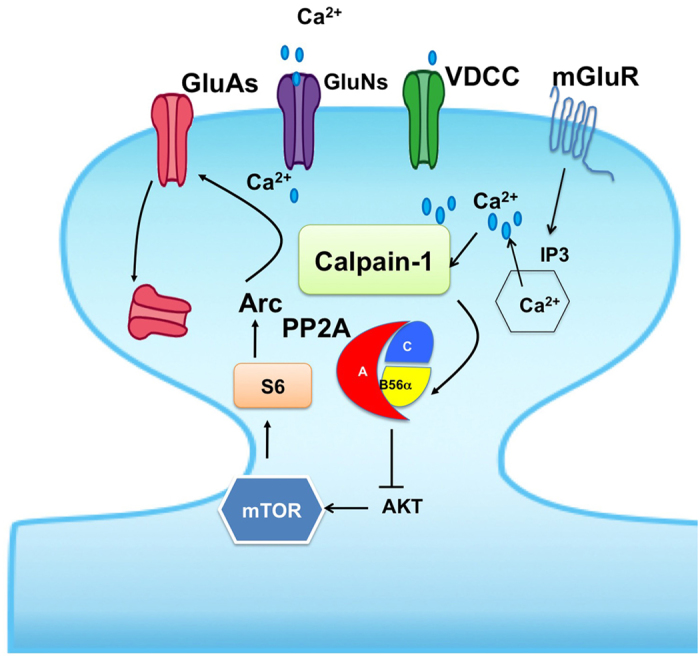
Schematic illustration of the potential mechanism underlying mGluR- LTD. In the proposed model, mGluR1/5 stimulation activates calpain-1 following calcium entry through T-type voltage dependent calcium channel. Calpain-1 degrades the B56α subunit of PP2A, which results in decreased PP2A activity and stimulation of the Akt/mTOR/S6 pathway, leading to increase in Arc and PSD95 synthesis. Increased synaptic levels of Arc and PSD95 facilitate endocytosis of GluA1-containing AMPA receptors, producing LTD. In calpain-1 KO mice, the lack of B56α degradation in response to mGluR1/5 stimulation prevents activation of this signaling pathway, which results in LTD impairment. Application of a PP2A inhibitor substitutes for calpain-1 activation, and therefore reverses the impairment in LTD and the associated changes in Arc and PSD95.

Extrasynaptic NMDA receptor activation is thought to mediate LFS-LTD^42^,^43^. In support of this idea, LFS-induced LTD is enhanced in some disease models in which extrasynaptic NMDA receptors are overactivated^38^. Recent work from our laboratory indicates that calpain-2 but not calpain-1 is activated by extrasynaptic NMDA receptor activation^31^, which is consistent with our finding that NMDAR-LTD was normal in calpain-1 KO mice. We also found that calpain-2 limits the magnitude of TBS-induced LTP^25^, although whether this calpain isoform is involved in NMDAR-LTD remains to be addressed. As opposed to calpain-2, calpain-1 is recruited to synaptic NMDA receptor protein complex upon receptor stimulation and promotes neuronal survival through cleavage of PHLPP1α^31^, an Akt phosphatase. However, this signaling pathway was not activated by mGluR1/5 stimulation, as PHLPP1α levels remained unchanged after DHPG treatment. Likewise, mGluR1/5 activation did not affect the levels of PHLPP1β, a negative regulator of ERK that is specifically cleaved by calpain-1 during LTP induction^25^. These results are consistent with the fact that DHPG-induced ERK phosphorylation was normal in calpain-1 KO mice. Nevertheless, our data indicate that mGluR1/5 stimulation specifically activates calpain-1 but not calpain-2, as DHPG treatment results in increase in SBDP levels in WT mice but not in calpain-1 KO mice. In contrast, the levels of the specific calpain-2 substrate, PTEN^32^, were not affected by DHPG treatment in either WT or calpain-1 KO mice. DHPG application also resulted in a rapid and calpain-1 dependent decrease in B56α, the regulatory subunit of PP2A, which was previously shown to be a calpain-1 substrate^35^. Collectively, these findings indicate that stimulation of mGluR1/5 triggers calpain-1 but not calpain-2 activation in some subcellular compartments distinct from those associated with the synaptic NMDA receptor complex. These results also suggest that calpain-1 and -2 activities and substrate specificities are tightly regulated by different glutamate receptors, possibly via formation of unique scaffold complexes, which in turn selectively activate specific signaling cascades. This idea is further supported by the presence of different types of PDZ binding domains at the C-terminal region of calpain-1 and -2^39^. These data are schematically represented in Fig. 7, which represents our proposed mechanism to link activation of mGluR1/5 to AMPA receptor internalization and LTD.

mGluR-LTD required the activation of T-type but not L-type VDCC or IP_3_ receptor. Similar findings were obtained in rats using LFS in the presence of an NMDA receptor antagonist to induce mGluR-LTD in CA1 region^18^. While mGluR-LTD in slices from neonatal mice appears to be independent of calcium influx, and maintained by presynaptic modifications, it becomes protein synthesis-dependent and maintained by postsynaptic CamKII in slices from more mature rodents^21^,^22^. The role of postsynaptic calcium in mGluR-LTD in slices from adult rats or mice has not been clearly demonstrated, but our results strongly suggest that T-type calcium channels and calpain-1 are involved. Differences in the various protocols used to induce mGluR-LTD could account for the various results obtained in different laboratories. In particular, we used a combination of very low frequency stimulation of CA1 afferents with DHPG application to induce LTD, which could affect the signaling pathways activated.

PP2A has been previously implicated in mGluR-LTD through the rapid (within 1 min) dephosphorylation of FMRP^29^, thereby releasing inhibition of Arc protein translation^39^. Interestingly, this activation was followed by a decrease in PP2A activity 2–5 min after DHPG application, an effect attributed to mTOR-mediated phosphorylation/inhibition^29^. However, in that study, PP2A activity also declined in the presence of rapamycin 10 min after DHPG stimulation, indicating that other mechanisms were involved. Our findings suggest that the decrease in PP2A activity is more likely due to calpain-1-mediated cleavage of the B56α subunit of PP2A, which was observed as early as 5 min and lasted up to 60 min after DHPG application. B56 subunits of PP2A (including B56α) have been shown to regulate ERK and Akt phosphorylation, both at Thr308 and Ser473^33^,^34^. Under our experimental conditions, Akt appeared to be preferentially targeted by PP2A, as DHPG-induced Akt but not ERK phosphorylation was impaired in calpain-1 KO mice. In support of this notion, PP2A is more tightly associated to Akt in mouse embryonic fibroblasts deficient in calpain-1, as compared to WT^35^. Nevertheless, considering that S6 kinase ß-1 (S6K1) is also a substrate of PP2A^44^, calpain-1-mediated B56α truncation could also directly contribute to S6K1 activation in mGluR-LTD. In any event, our findings strongly suggest that calpain-1-mediated degradation of PP2A subunit B56α contributes to DHPG-induced Akt phosphorylation and subsequent activation of mTOR/S6-dependent protein synthesis.

Local translation of Arc (among other proteins) has been repeatedly shown to be involved in mGluR-LTD^37^,^38^,^39^,^45^. However, while several proteins have been implicated in the early rise in Arc synthesis, including eEF2K and FMRP^37^,^39^, it is not clear yet which signaling cascades and translation factors mediate the late phase of Arc translation in response to DHPG application. Our results indicate that calpain-1 mediated activation of the Akt/mTOR/S6 pathway is responsible for the maintenance of Arc translation during mGluR-LTD. This conclusion is based on the facts that DHPG-induced late Arc synthesis was blocked by rapamycin and was also absent in slices from calpain-1 KO mice, but was restored by phosphatase inhibition in these slices. Consistent with these results, studies from different groups have demonstrated that activation of the Akt/mTOR signaling pathway was required for mGluR-LTD^15^,^46^. Although mGluR-LTD was still expressed or enhanced in mice lacking S6K1 and/or S6K2^28^, it is possible that other translation factors downstream of mTOR such as 4EBP2 could compensate for the loss of S6K1/2 and support Arc synthesis and mGluR-LTD^46^,^47^. In addition, transcription and transport of Arc mRNA to dendrites may also contribute to the late phase of Arc synthesis according to studies performed in cultured neurons^37^,^48^. Overall, the activation of Akt/mTOR/S6 pathway seems to act in concert with other signaling proteins, including CaMKII, eIF2α, eEF2K and ERK^14^,^22^,^30^,^37^ to regulate protein translation in mGluR-LTD. Importantly, while many of these signaling pathways are required for mGluR-LTD, none of them appears to be sufficient to support it alone^46^. Our results differ from recent results indicating that glutamate-induced changes in Arc synthesis in cultured cortical neurons are not blocked by rapamycin, suggesting that another pathway is involved in the regulation of Arc synthesis^49^. These differences could be due to the different systems under investigation and the different protocols used to elicit changes in Arc synthesis.

Phosphorylation and dephosphorylation of AMPA receptors, especially of GluA1 subunits, have been proposed to contribute to the expression of LTP and LTD, respectively^50^. Internalization of AMPARs has also been shown to mediate synaptic depression during mGluR-LTD, a process that requires local protein synthesis^38^,^50^. Consistent with these findings, we found that DHPG-induced decrease in GluA1 was blocked by the translation inhibitor cycloheximide. In addition, GluA1 degradation by lysosomes and by the ubiquitin-proteasome system has been recently associated with NMDAR-LTD and mGluR-LTD, respectively^13^,^51^,^52^. Our results showing decreased GluA1 levels in response to DHPG application both by immunostaining and by immunoblot in whole hippocampal homogenates support the notion that GluA1-containing AMPA receptors undergo rapid degradation following DHPG treatment. The reduction in GluA1 levels was dependent on calpain-1 activation, but was not related to direct GluA1 truncation by calpain-1, which was evidenced by decreased GluA1 levels labeled with a C-terminal antibody without a shift in GluA1 molecular weight using an N-terminal antibody, which would have occurred since calpain-mediated GluA1 truncation takes place in the C-terminal domain^53^. Thus, our results indicate that calpain-1 activation is necessary for DHPG-induced GluA1 internalization, as a result of Arc synthesis elicited by stimulation of the Akt/mTOR/S6 signaling pathway.

Noteworthy, PSD95 staining was rapidly increased after DHPG application, an effect blocked by protein synthesis inhibitors. These findings are in good agreement with previous studies that reported a rapid increase in local synthesis of PSD95 in response to mGluR1/5 activation^54^,^55^,^56^. Yet, the precise role of PSD95 translation in mGluR-LTD still remains elusive. In this regard, PSD95 was shown to be required for GluA1 internalization during NMDA- but not mGluR-LTD^57^. Therefore, additional studies are needed to elucidate the function of PSD95 in mGluR-LTD.

Activation of mGluR1/5 has previously been proposed to be involved in extinction of fear conditioning as well as in reversal learning^4^,^58^,^59^,^60^. As calpain-1 KO mice are impaired in both mGluR-LTD and fear extinction, it is tempting to hypothesize that mGluR-LTD may play a role in extinction of fear memories. However, the mechanisms involved in fear extinction are still debated^61^. In particular, it has been suggested that extinction might reverse learning-induced changes (depression or depotentiation) or enhance inhibitory synaptic transmission (potentiation) in amygdala^61^. While our results indicate that mGluR-LTD is impaired in field CA1 of hippocampus, we have no evidence that a similar process takes place in amygdala. Likewise, although we showed that TBS-LTP was impaired in calpain-1 KO mice^24^, we do not know whether calpain-1 is also involved in LTP at inhibitory GABAergic synapses. Further studies are needed to address these different possibilities.

In summary, our findings indicate that calpain-1 activation participates in different forms of plasticity, including TBS-LTP and mGluR-LTD. In each case, the mechanisms of calpain-1 activation are different (synaptic NMDA receptor versus T-type calcium channels) as are the downstream signaling pathways (PHLPP1 versus PP2A). Such a diversity of signaling is likely due to the association of calpain-1 with different clusters of proteins through selective protein-protein interactions^39^. We recently reported that calpain-1 mutations/deletion are associated with cerebellar ataxia in mice and humans^62^, and it will be of interest to determine whether the mutations in humans are also associated with impairment in extinction and other forms of associative learning.

## Materials and Methods

### Reagents

(RS)-3,5-Dihydroxyphenylglycine (DHPG), NNC 55–0396 dihydrochloride (NNC), nimodipine, 2-aminoethoxydiphenylborane (2-APB) and cycloheximide were obtained from Tocris. Rapamycin and Calyculin-A were from Cell Signaling. Calpain inhibitor III was from Calbiochem. Okadaic acid was from Santa Cruz. All the other reagents were from Sigma. Water insoluble compounds were dissolved in DMSO and diluted to reach a final concentration less than 0.1%.

### Animals

All experiments followed NIH guidelines for the use of experimental animals and all protocols were approved by the Institution Animal Care and Use Committee of Western University of Health Sciences. Calpain-1 KO mice on a C57Bl/6 background were obtained from a breeding colony established from breeding pairs generously provided by Dr. Chishti (Tufts University). C57Bl/6 mice were purchased from Jackson Labs and were used as the corresponding wild type (WT) mice.

### Acute hippocampal slice preparation

Hippocampal slice preparation was performed as previously reported^25^. Briefly, adult male mice (3–4-month-old) were anesthetized with halothane and decapitated. Brains were quickly removed and transferred to oxygenated, ice-cold cutting medium (in mM): 124 NaCl, 26 NaHCO_3_, 10 glucose, 3 KCl, 1.25 KH_2_PO_4_, 5 MgSO_4_, and 3.4 CaCl_2_. Hippocampal transversal slices (350 μm-thick) were prepared using a McIlwain-type tissue chopper and transferred to an interface recording chamber and exposed to a warm, humidified atmosphere of 95% O_2_/5% CO_2_ and continuously perfused with oxygenated and preheated (33 ± 0.5 °C) aCSF (in mM) [110 NaCl, 5 KCl, 2.5 CaCl_2_, 1.5 MgSO_4_, 1.24 KH_2_PO_4_, 10 D-glucose, 27.4 NaHCO_3_] at a speed of 1.4 ml/min.

### Electrophysiology

After 2 h of incubation at 33.0 ± 0.5 °C in recording chamber, a single glass pipette filled with 2 M NaCl was used to record field EPSPs (fEPSPs) elicited by stimulation of the Schaffer collateral pathway with twisted nichrome wires (single bare wire diameter, 50 μm) placed in CA1 stratum radiatum. Responses were recorded through a differential amplifier (DAM 50, World Precision Instruments, USA) with a 10 kHz high-pass and 0.1 Hz low-pass filter. Before each experiment, the input/output (I/O) relation was examined by varying the intensity of the stimulation. We previously reported that the I/O curve in slices from calpain-1 KO mice was slightly different from that in slices from WT mice at high intensities of stimulation^24^. For all experiments, we selected a stimulus intensity eliciting 60% of the maximum response, and under these conditions, baseline responses were not different in slices from calpain-1 KO and WT mice. LTD was induced by (i) application of DHPG (100 μM, 10 min), (ii) delivering low frequency paired stimulation (50 ms interval, 1 Hz, 15 min) in the presence of the NMDA receptor antagonist, AP5, or (iii) low frequency stimulation (1 Hz, 15 min). Data were collected and digitized by Clampex, and the slope of fEPSP was analyzed. LTD level was normalized to the 10 min baseline.

### Western blotting

After treatments, slices were collected in dry ice and homogenized in lysis buffer (50 mM Tris-HCl, pH 7.4, 150 mM NaCl, 1% Triton) containing protease and phosphatase inhibitor cocktail (Thermo). Protein concentrations were measured using the BCA protein assay kit (Thermo). Equal amounts of proteins (20–30 μg) were processed for SDS-PAGE and western blot, as previously described^63^. The primary antibodies used were PTEN (1:1000, Cell signaling), phospho-ERK T202/Y204 (1:2000, Cell Signaling), ERK (1:3000, Cell signaling), phospho-Akt S473 (1:1000, Cell signaling), Akt (1:2000, Cell signaling), phospho-S6 S240/S244 and S6 (both 1:1000, Cell signaling), phospho-eIF2α S51 (1:500, Cell signaling), eIF2α (1:1000, Cell signaling), PP2A B56 alpha subunit (1:200, Santa Cruz), GLUA1 N-terminal (1:200, Santa Cruz), GLUA1 C-terminal (1:1000, Millipore), α-spectrin (1:2000, Millipore), PHLPP1 (1:1000, Millipore), actin (1:10000, Millipore).

### Immunohistochemistry

Immunostaining was performed in frozen sections from hippocampal slices as described previously^25^. Antibodies: rabbit anti-Arc (1:500, Millipore), rabbit anti-GLUA1 (1:400, AB1504, Millipore) and mouse anti-PSD95 (1:500, MA1-045, Thermo Scientific). Image acquisition and analysis were performed as described previously^13^.

### Fear conditioning and extinction

Mice were housed individually with normal 12/12 h daylight cycle. They were handled daily for 5 days prior to training. On training day, they were placed in the fear-conditioning chamber (H10-11M-TC, Coulbourn Instruments) located in the center of a sound-attenuating cubicle (Coulbourn Instruments). The conditioning chamber was cleaned with 10% ethanol to provide a background odor. A ventilation fan provided a background noise at ∼55 dB. After a 2 min exploration period, three tone-footshock pairings separated by 1 min intervals were delivered. The 85 dB 2 kHz tone lasted for 30 s, and the footshocks were 0.75 mA and lasted for 2 s. Footshocks co-terminated with the tone. Mice remained in the training chamber for another 30 s before being returned to their home cages. Extinction trials started the following day and were repeated two more days. The same conditioning chamber was modified by changing its metal grid floor to a plastic sheet, white metal walls to plastic walls gridded with red tapes, and odor from ethanol to acetic acid. The ventilation fan was turned off to reduce background noise and the ceiling light was changed from yellow to white. Each day, mice were placed in the chamber for 2 min, and the tone was presented for 5 s for 14 trials with an inter-trial interval of 25 s. Mice behavior was recorded with the Freezeframe software and analyzed with Freezeview software (Coulbourn Instruments). Motionless bouts lasting more than 1 s were considered as freezing. The percent of freezing time following each tone presentation was determined and the cumulative percent freezing time per day determined and averaged.

### Statistical analyses

Data are presented as means ± SEM. For experiments where only two groups were compared, two-tail *t*-test was used for determining statistical significance. When more than two groups were compared, we used one-way or two-way ANOVA followed by Bonferroni post-hoc test to determine statistical significance. p values less than 0.05 were considered statistically significant.

## Additional Information

**How to cite this article:** Zhu, G. *et al*. Calpain-1 deletion impairs mGluR-dependent LTD and fear memory extinction. *Sci. Rep.* **7**, 42788; doi: 10.1038/srep42788 (2017).

**Publisher's note:** Springer Nature remains neutral with regard to jurisdictional claims in published maps and institutional affiliations.

## Supplementary Material

Supplementary Information
