# Supplementary material for: Calpain-1 deletion impairs mGluR-dependent LTD and fear memory extinction
Source: Sci Rep. 2017 Feb 16;7:42788. doi: 10.1038/srep42788 (PMC5311935; doi:10.1038/srep42788)
Supplement: Supplementary Information [file srep42788-s1.doc]

**Calpain-1 deletion impairs mGluR-dependent LTD and fear memory extinction**

Guoqi Zhu^1,3*^, Victor Briz^1,4*^, Jeff Seinfeld^1^, Yan Liu^1,2^, Xiaoning Bi^2^ and Michel Baudry^1^

**Supplementary Data**

**
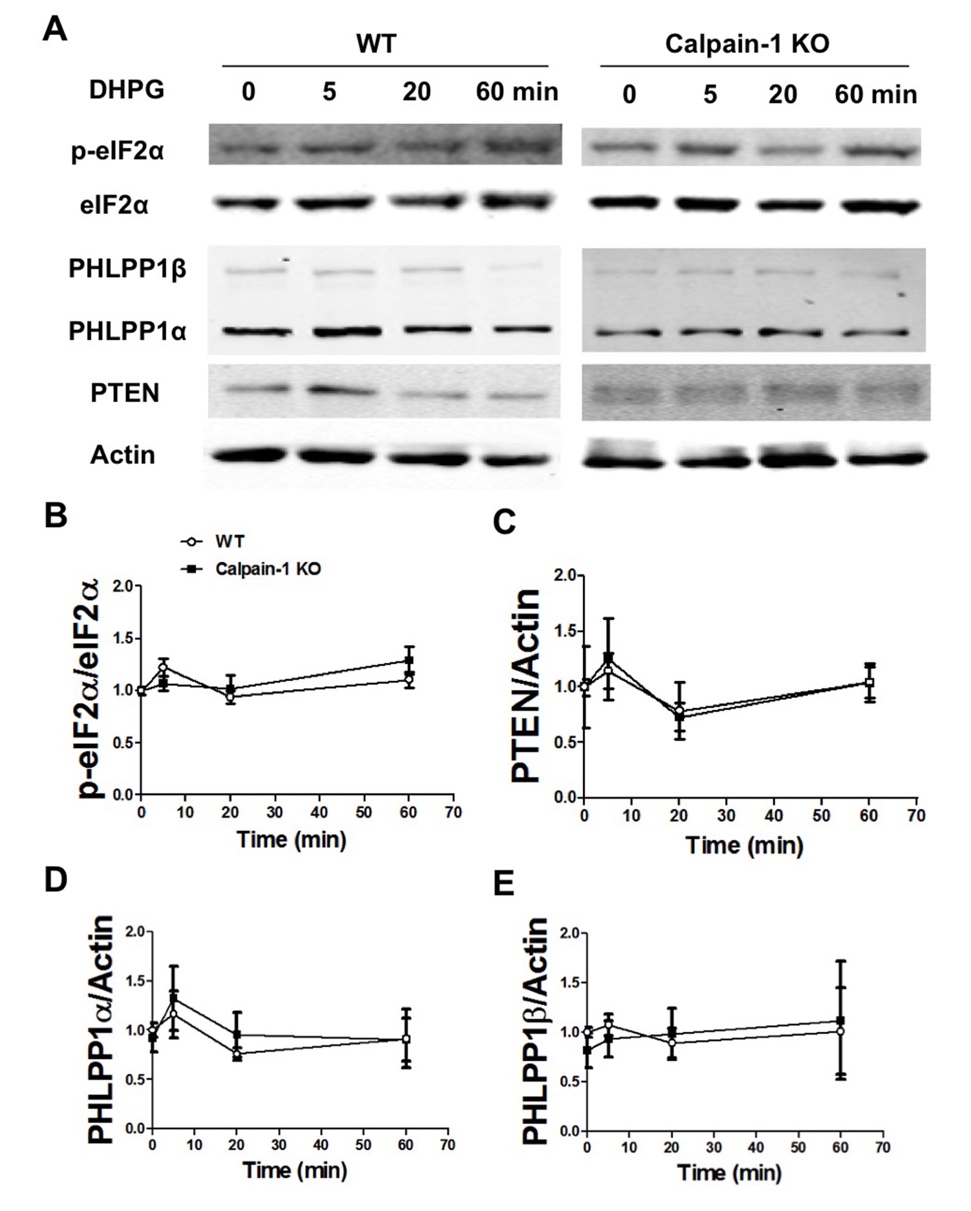
**

**Figure S1: Effects of DHPG application on eIF2a phosphorylation and PHLPP1 and PTEN levels.**

Hippocampal slices from WT and calpain-1 KO mice were treated with DHPG (100 µM, 10 min). Slices were collected at various times after DHPG application, homogenized and aliquots of the homogenates were processed for western blots labeled with the indicated antibodies. **A.** Representative blots. **B-E.** Quantification of the ratio of phospho-eIF2α (p-eIF2α)/eIF2α (**B**), PTEN/actin (**C**), PHLPP1α/actin (**D**), PHLPP1β/actin (**E**). In all cases, results are means ± S.E.M. of 3-7 slices from 3 different animals.


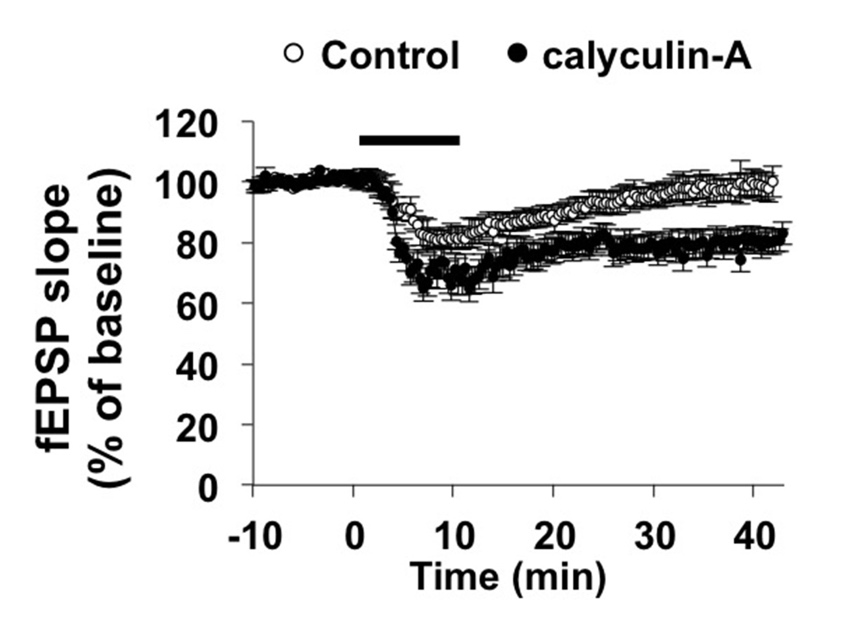


**Figure S2:** **The** **PP2A inhibitor calyculin-A restores mGluR-LTD in calpain-1 KO mice.**


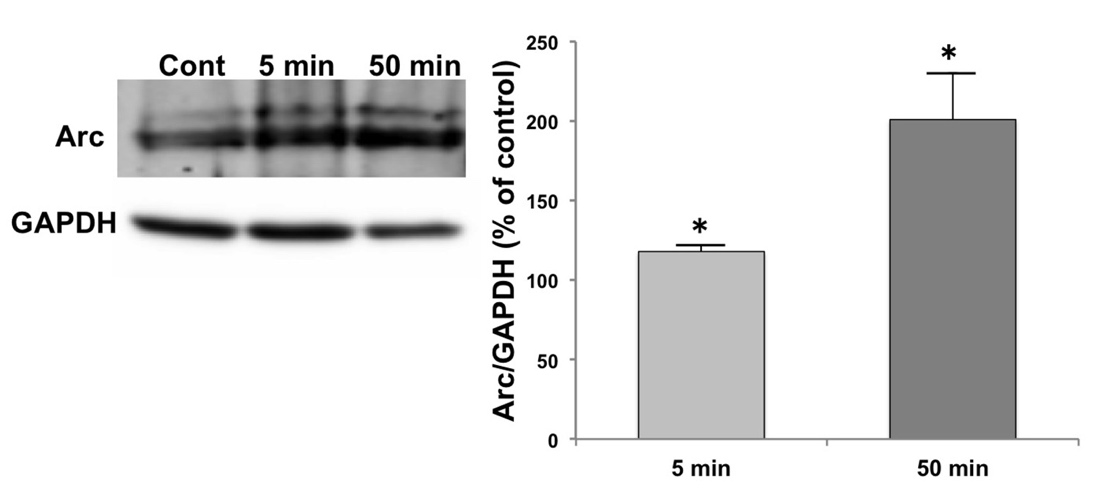
The PP2A inhibitor calyculin-A (10 nM) restored LTD induced by DHPG application (100 µM, 10 min, horizontal bar) in hippocampal slices from calpain-1 KO mice. Results are means ± S.E.M. of 3-7 slices from 3-5 animals.

**Figure S3: mGluR-LTD is associated with increased levels of Arc.**

Hippocampal slices from WT mice were treated with DHPG (100 µM, 10 min). Slices were collected at the indicated times after DHPG application, homogenized and aliquots of the homogenates were processed for western blots labeled with the indicated antibodies. **A.** Representative blots. **B.** Quantification of the ratio of Arc/GAPDH. Results are means ± S.E.M. of 3 slices from 2 different mice. * p< 0.05, Student’s t-test.


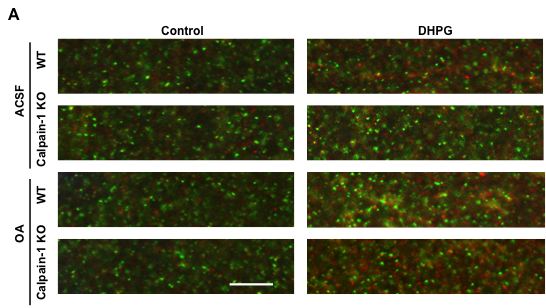


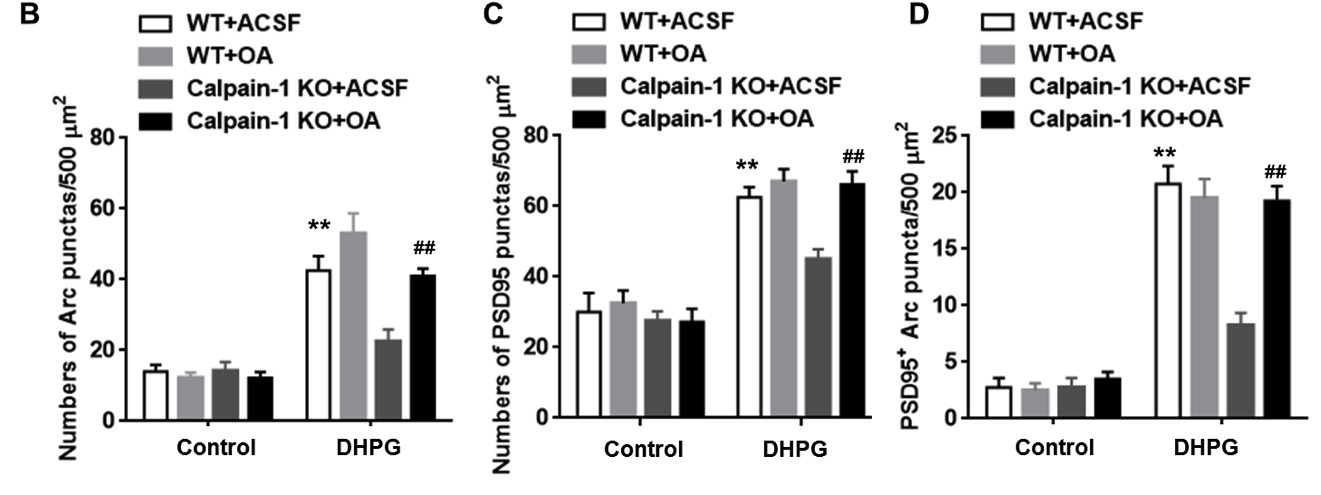


**Figure S4:** **Okadaic acid rescued the impairment of DHPG-induced Arc expression in calpain-1 KO mice.**

DHPG application (100 µM for 10 min) induced a significant increase in Arc levels in apical dendrites of CA1 hippocampal neurons 50 min after DHPG application. A) Upper panel shows representative images of dendritic Arc and PSD95 immunostaining. Red: Arc, Green: PSD95; scale bar: 20 µm. **B.** Quantification of the numbers of Arc-positive punctas; **p<0.01, as compared with baseline level in WT mice; ^##^p<0.01 compared with DHPG in calpain-1 KO mice (Two-way ANOVA + Bonferroni test). **C.** Quantification of the numbers of PSD95-positive punctas; **p<0.01, as compared with baseline level in WT mice; ^##^p<0.01 compared with DHPG in calpain-1 KO mice (Two-way ANOVA + Bonferroni test). **D**. Quantification of the PSD95 and Arc double-positive punctas; **p<0.01, as compared with baseline level in WT mice; ^##^p<0.01 compared with DHPG in calpain-1 KO mice (Two-way ANOVA + Bonferroni test). Results are means ± S.E.M. of 3-7 slices from 3-5 animals.


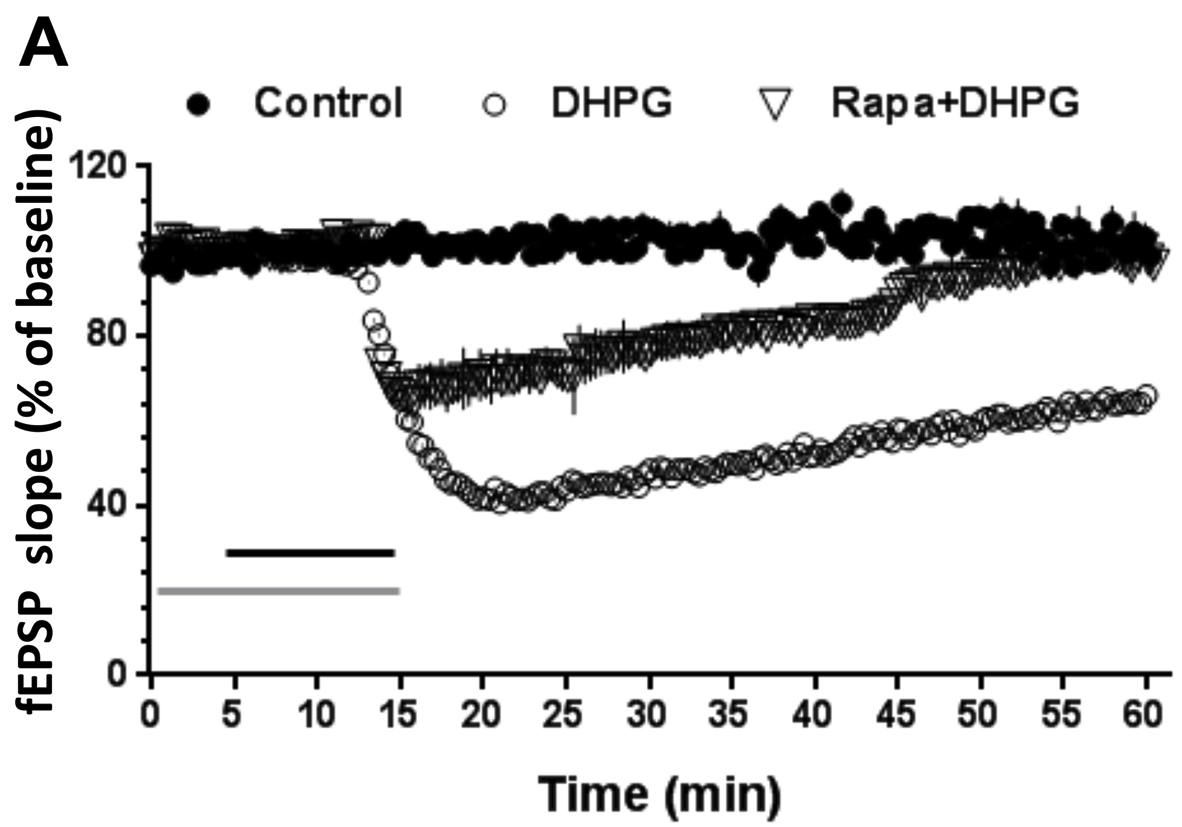


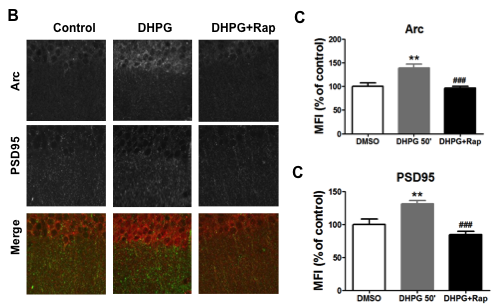


**Figure S5:** **DHPG-induced LTD and late translation of Arc and PSD95 requires mTOR activation.**

**A.** Pre-incubation with rapamycin (Rap, 20 nM, 30 min) abolished DHPG-induced LTD 100 µM for 10 min, horizontal bar). Control: closed circles; DHPG: open circles; DHPG + rapamycin: open triangles. Results are means ± S.E.M. of 3-4 slices.. **B.** Arc and PSD95 immunostaining of hippocampal slices 50 min following treatment with vehicle (Control, DMSO), DHPG (100 µM, 10 min) or Rapamycin + DHPG. Scale bar = 20 µm. **C.** Quantification of the data shown in B. Results are expressed as percent mean fluorescence intensity (MFI) present in control slices and are means ± S.E.M. of 3-7 slices from 3-5 animals
